# Pathophysiology, Histopathology, and Differential Diagnostics of Basal Cell Carcinoma and Cutaneous Squamous Cell Carcinoma—An Update from the Pathologist’s Point of View

**DOI:** 10.3390/ijms25042220

**Published:** 2024-02-13

**Authors:** Iuliu Gabriel Cocuz, Maria Cătălina Popelea, Raluca Niculescu, Andrei Manea, Adrian-Horațiu Sabău, Andreea-Cătălina Tinca, Andreea Raluca Szoke, Corina Eugenia Budin, Adina Stoian, Silviu Horia Morariu, Titiana Cornelia Cotoi, Maria-Elena Cocuz, Ovidiu Simion Cotoi

**Affiliations:** 1 Pathophysiology Department, “George Emil Palade” University of Medicine, Pharmacy, Sciences and Technology of Targu Mures, 540142 Targu Mures, Romania; iuliu.cocuz@umfst.ro (I.G.C.); raluca.niculescu@umfst.ro (R.N.); adrian-horatiu.sabau@umfst.ro (A.-H.S.); andreeatinca93@gmail.com (A.-C.T.); szoke.andreea@yahoo.com (A.R.S.); cora_bud@yahoo.com (C.E.B.); adina.stoian@umfst.ro (A.S.); ovidiu.cotoi@umfst.ro (O.S.C.); 2 Pathology Department, Mures Clinical County Hospital, 540011 Targu Mures, Romania; 3 Faculty of Medicine, “George Emil Palade” University of Medicine, Pharmacy, Sciences and Technology of Targu Mures, 540142 Targu Mures, Romania; andrei17v1997@icloud.com; 4 Pneumology Department, Mures Clinical County Hospital, 540011 Targu Mures, Romania; 5 Neurology I Clinic, Targu Mures Emergency County Hospital, 540136 Targu Mures, Romania; 6 Dermatology Department, “George Emil Palade” University of Medicine, Pharmacy, Sciences and Technology of Targu Mures, 540142 Targu Mures, Romania; silviu_morariu@yahoo.com; 7 Dermatology Department, Mures Clinical County Hospital, 540011 Targu Mures, Romania; 8 Pharmaceutical Technique Department, “George Emil Palade” University of Medicine, Pharmacy, Sciences and Technology of Targu Mures, 540142 Targu Mures, Romania; titiana.cotoi@umfst.ro; 9 Pharmacy No. 2, Mures Clinical County Hospital, 540011 Targu Mures, Romania; 10 Fundamental Prophylactic and Clinical Disciplines Department, Faculty of Medicine, Transilvania University of Brasov, 500003 Brașov, Romania; maria.cocuz@unitbv.ro; 11 Clinical Pneumology and Infectious Diseases Hospital of Brasov, 500174 Brasov, Romania

**Keywords:** non-melanocytic skin cancers, basal cell carcinoma, cutaneous squamous cell carcinoma, histopathology, skin cancer, immunohistochemistry

## Abstract

Basal cell carcinoma (BCC) and cutaneous squamous cell carcinoma (cSCC) are the most frequently occurring non-melanocytic skin cancers. The objective of our study is to present the pathophysiology of BCC and cSCC and its direct relationship with the histopathological diagnostics and the differential diagnostics of these types of cancer, based on the morphological characteristics, immunohistochemical profile, and genetic alterations. The qualitative study was based on emphasizing the morphological characteristics and immunohistochemistry profiles of BCC and cSCC and the differential diagnostics based on the tissue samples from the Clinical Pathology Department of Mures Clinical County Hospital between 2020 and 2022. We analyzed the histopathological appearances and immunohistochemical profiles of BCC and cSCC in comparison with those of Bowen disease, keratoacanthoma, hyperkeratotic squamous papilloma, metatypical carcinoma, pilomatricoma, trichoblastoma, Merkel cell carcinoma, pleomorphic dermal sarcoma (PDS), and melanoma. Our study showed the importance of the correct histopathological diagnosis, which has a direct impact on the appropriate treatment and outcome for each patient. The study highlighted the histopathological and morphological characteristics of NMSCs and the precursor lesions in HE and the immunohistochemical profile for lesions that may make the differential diagnosis difficult to establish.

## 1. Introduction

Non-melanocytic skin cancers (NMSCs) represent a worldwide public health problem due to their risk factors and increased incidence. Within the framework of this study, the approach to these particular types of cancer, i.e., basal cell carcinoma (BCC) and cutaneous squamous carcinoma (cSCC), represents a structure based on two axes—a medical axis and a research axis—of a multidimensional approach to their management, diagnosis, and treatment.

The microscopic interpretation of the histopathological preparations is based on the histopathological slides that result from the sampling of biological tissue, its inclusion in paraffin, and its staining with the usual Hematoxylin–Eosin stain. To establish certain diagnoses in particular situations, immunohistochemical reactions can be performed.

The 2018 WHO skin tumor classification defines non-melanocytic skin cancers as keratinocyte malignancies derived from the epidermis [1].

The main risk factors for BCC are divided into eight categories, each of which modifies the physiological structure of DNA, with direct effects on transcription [2]: age of patients, sex (gender) of patients, exposure to UV radiation (professional/recreational), skin phototype, genetic syndromes, immunodepression, pharmacological therapy and radiotherapy, and family history of skin tumors.

The main risk factors for the development of cSCC are represented by seven categories: age of patients, sex (gender) of patients, exposure to UV radiation (professional/recreational), skin phototype, immunosuppression, genetic syndromes, and viral infections [3,4].

### 1.1. Pathogenesis of BCC

The pathophysiological mechanism of BCC development is very complex and involves the interaction of various factors, including genetic factors and environmental factors. The genetic changes involved in the pathogenesis of BCC are related to DNA damage from ultraviolet rays [2,3,4,5]. 

The classical Hedgehog pathway is involved in the control of the cellular functions necessary for the activity of various tissues, such as skin, bone, or intestinal tissue [6,7,8]. The impairment of the Hedgehog signaling pathway can have serious implications when it is hyperactivated or hypoactivated. Thus, during embryogenesis, the hypoactivation of the Hedgehog pathway can cause birth defects, and hyperactivity during adult life leads to carcinogenesis [9,10]. Changes in the HH pathway represent the most common pathophysiological mechanism in the development of BCC. During embryogenesis, the HH pathway has a particularly important role in the development of the epidermis and hair follicles [11].

### 1.2. Pathogenesis of cSCC

Multiple signaling pathways are involved in the development of cSCC and play defined roles in the pathogenesis of these lesions. Starting from precursor lesions, such as actinic keratosis (AK) or Bowen disease (BD), all the changes that lead to the appearance of cSCC involve a series of mutations in normal cellular homeostasis that begins with the uncontrolled proliferation of keratinocytes. At the same time, a number of other factors, such as epigenetic factors, viral infections, or changes in the microclimate, favor the development and progression of cSCC [12,13]. AK is the most frequent precursor lesion that may evolve into cSCC; it is considered by some authors to be a subtype of Bowen disease [14].

The most frequent genetic mutations in the development and pathogenesis of cSCC are represented by pathways involved in cell cycle regulation and programmed cell death (apoptosis) or signaling pathways related to senescence, differentiation, or mitotic proliferation [15].

The TP53 gene represents the most frequent mutation involved in the pathogenesis of cSCC. TP53 encodes the suppressor protein p53, which has an important role as a transcription factor in maintaining genome stability. One of the many roles that p53 plays involves the intervention regarding the arrest and control of the cell cycle, apoptosis, DNA repair, senescence, or changes in metabolism. In the pathogenesis of cSCC, the inactivation of the p53 protein, which is caused by either genetic mutations or viral infections, such as HPV (human papilloma virus) infection, is implicated [16,17,18].

The EGFR gene has a major involvement in the development of cSCC in terms of mitotic activity. The increased expression of EGFR and the activation of the EGFR pathway are observed in 43–83% of cSCC cases [17,19,20,21].

The HPV virus is a DNA virus that targets the squamous epithelium. Thus, serological subtypes 16 and 18 are directly involved in the oncogenesis of SCC (at the oropharyngeal and anogenital levels) and also that of cSCC. By engaging the E6 and E7 proteins of the HPV virus, it affects the p53 pathway and the retinoblastoma pathway, inducing uncontrolled tumor proliferation [16,22,23,24,25,26,27,28,29]. 

The tumoral microclimate is of particular importance in the development of cSCC, as it is made up of a complex of molecules and cells involved in tumor proliferation. The interaction between normal keratinocytes and the cells present in the tumor microclimate leads to impairment of the epigenetic pathways [16,25,26,27,28,29,30].

### 1.3. Immunohistochemical Profile of cSCC

Immunohistochemically, the tumor cells in BCC are positive for immunolabeling with anti-Bcl-2, BerEP4 antibodies, and anti-CD10 antibodies. The immunohistochemistry of BCC is not used for routine diagnosis. It is used more in the differential diagnosis of these tumors and metatypical carcinomas or in research [1,7].

### 1.4. Immunohistochemical Profile of cSCC

Immunohistochemically, the tumor cells in cSCC are positive for immunolabeling with anti-p63, anti-p40, epithelial membrane antigen (EMA), anti-CK5/6, and panCK/CK AE1/AE3 antibodies (a cytokeratin cocktail with a high molecular weight) [1,7,31,32]. 

The objective of our study is to present the pathophysiology of BCC and cSCC and its direct relationship with the histopathological diagnostics and the differential diagnostics of these types of cancer, based on the morphological characteristics, immunohistochemical profile, and genetic alterations.

## 2. Results and Discussion

Non-melanocytic skin cancers present a variety of different histopathological features and immunohistochemical profiles. Starting from the individual characteristics of each non-melanocytic lesion, their microscopic characteristics must be analyzed very carefully to establish the diagnoses of BCC and cSCC and their differential diagnosis. Table 1 presents the main characteristics of BCC and cSCC and their histopathological classifications.

### 2.1. Basal Cell Carcinoma

Any step that is impaired in the activation of the Hedgehog pathway leads to oncogenesis through an aberrant transcription. In the pathophysiological mechanism of BCC, the gene for PTCH1 is involved in the inactivation of the classical Hedgehog pathway. Most mutations of the PTCH1 gene are caused by UV radiation, leading to impaired transcriptions. SMO gene mutations and combined PTCH1 and SMO mutations have been observed in patients diagnosed with BCC [8,10,36].

GLI transcription factors have different functions depending on their type. Thus, the transcription factor GLI1 is involved in the activation of transcription, and GLI2 and GLI3 have both activatory and inhibitory functions in transcription. Any mutation that occurs within the transcription factors leads to the impairment of the normal cell proliferation process [36,37].

For the functioning of the HH pathway, the proper functioning of the mediators for cell proliferation and differentiation (Cyclin D1-D2) and for cell survival (Bcl-2—B-cell lymphoma 2), in addition to other factors, is necessary [8].

In addition to the classical HH pathway that is present in 85–90% of BCC cases, there are other mutations that can intervene in the HH pathway, such as mutations in the EGFR (epidermal growth factor receptor) and TGF-beta (transforming growth factor beta) genes [8].

**Figure 1:**
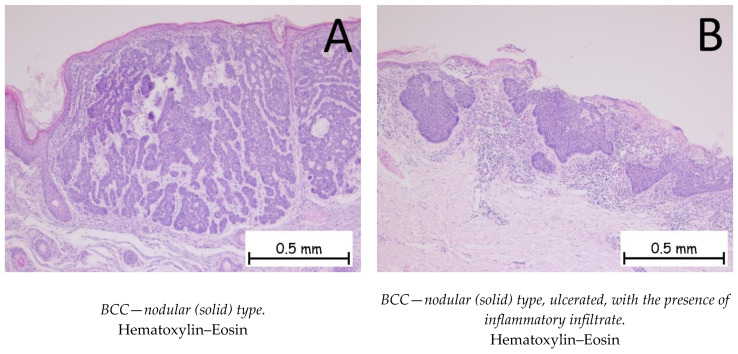
Basal cell carcinoma—nodular (solid) type. (**A**). Tegument covered by a keratinized stratified squamous epithelium, showing a tumoral proliferation in the underlying dermis composed of plaques of cells with a basaloid appearance, with nuclei palisading at the periphery. The plaques have a solid, compact appearance, with varied shapes and sizes. (**B**). Keratinized stratified squamous epithelium, ulcerated, showing a tumoral proliferation in the underlying dermis composed of plaques of cells with a basaloid appearance with abundant chronic inflammatory infiltrate in the peritumoral stroma.

**Figure 2:**
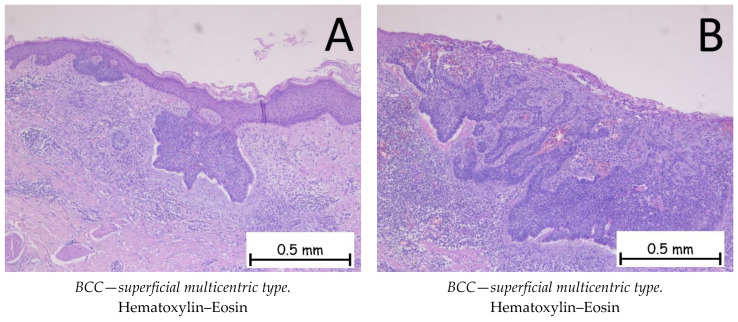
Basal cell carcinoma—superficial multicentric type. (**A**). Tegument covered by a keratinized, stratified squamous epithelium, ulcerated, intermittently presenting nests of basaloid-type tumor cells at the base of the epidermis with basophilic, reduced cytoplasm, large, hyperchromatic nuclei, and a palisade arrangement of nuclei at the periphery. (**B**). Ulcerated epidermis intermittently presenting nests of basaloid-type tumor cells at the base with peritumoral artifact retraction and an abundant chronic inflammatory infiltrate.

**Figure 3:**
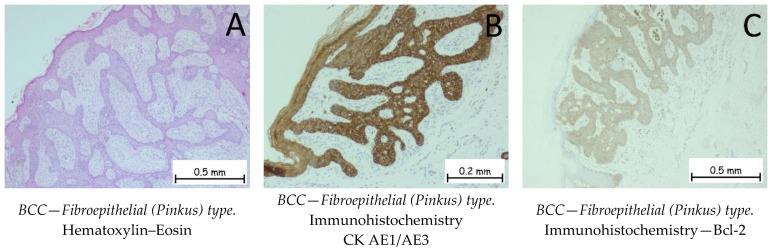
Basal cell carcinoma—fibroepithelial (Pinkus) type. (**A**). Interconnected strands and clusters of basaloid cells enveloped by a fibrous stroma. When viewed from a three-dimensional standpoint, the tumor takes on the appearance of a honeycomb or sponge, characterized by thin epithelial septa, with the intervening spaces filled by stroma. Along the edges of the fenestrations, columnar cells are organized in a palisade formation. The tumor cells are positive for anti-CK AE1/AE3 (**B**) and anti-Bcl-2 antibodies (**C**).

**Figure 4:**
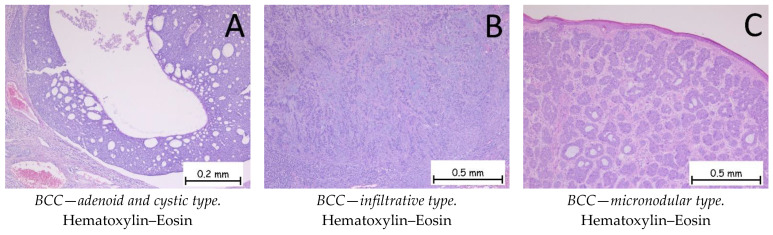
Basal cell carcinoma—adenoid and cystic type, infiltrative type, and micronodular type. (**A**). Adenoid and cystic type. Tumoral proliferation composed of plaques with pseudo-glandular appearance or with cystic degeneration. At the periphery of the plaques, nuclear palisading and artifact retraction of the surrounding stroma are observed. (**B**). Infiltrative type. Cords and trabeculae of basaloid-type tumor cells with basophilic, reduced cytoplasm, large nuclei, hyperchromatism, and a disposition resembling nuclear palisading at the periphery. The intertumoral stroma exhibits an abundant lymphoplasmacytic inflammatory infiltrate. (**C**). Micronodular type. Skin fragment covered by keratinized stratified squamous epithelium, presenting a tumoral proliferation in the underlying dermis composed of small, variable-shaped plaques with a solid, basaloid appearance.

*Basal cell carcinoma* is represented by a proliferation of cells from the basal layer of the epidermis. Depending on the possibility of recurrence, it is classified as a carcinoma with either a low risk or a high risk of recurrence. Figure 1, Figure 2, Figure 3 and Figure 4 show the histopathological features of BCC. Immunohistochemically, the tumor cells are positive for anti-Bcl-2 and anti-BerEP4 antibodies and for anti-CD10 antibodies. The differential diagnosis of basal cell carcinoma is generally made with trichoblastoma or cSCC with a basaloid differentiation. The genetic profile of basal cell carcinomas expresses mutations in the PTCH1 gene [1,38,39]. Depending on the subtype of BCC, the nodular subtype can be categorized as low risk in terms of a possible recurrence or metastasis, whereas the infiltrative subtype, due to its histopathological characteristics and risk of recurrence, is categorized as high risk. Consequently, the prognosis of BCC may be different, depending on the subtype [1,34].

### 2.2. Cutaneous Squamous Cell Carcinoma

There is a well-proven connection between UV radiation, especially UVB radiation, and the induction of mutations in the p53 gene. Thus, the interaction between UVB and the p53 gene has been demonstrated through numerous animal experiments [16,17]. LOF mutations in the Trp53 gene can alter the control of the cell cycle checkpoint, with a direct impact on the evolution of cSCC. [18].

TP53 sequence mutation occurs in 54–95% of cSCC cases, and due to its physiological role, this mutation is responsible for the increased genomic instability of these cancers. These mutations have been reported in precursor lesions (AK and BD) and in primary cSCC tumors and metastatic cSCC tumors [6,40,41]. 

The CDKN5A (cyclin-dependent kinase inhibitor 5A) locus encodes two proteins: p16INK4a and p14ARF. These proteins have an important role in inhibiting cell proliferation through the two pathways they control—the retinoblastoma pathway (pRb) and the p53 pathway. The p16INK4a protein is considered to be a tumor suppressor gene due to the fact that it inhibits the kinase activity of cyclin-dependent kinases (CDKs), thereby blocking the retinoblastoma pathway and the p53 pathway, which leads to the control of the cell cycle and cell senescence. The p14ARF protein is also a tumor suppressor gene whose role is to block MDM2 (MDM2 Proto-Oncogene) from forming a stable complex. Thus, the p53 pathway manages to function physiologically and to control the cell cycle [6,42,43].

Retinoblastoma is a malignant tumor proliferation of the retina. Retinoblastoma is caused by a tumor suppressor gene (Rb1 gene) that stabilizes heterochromatin. The mutation of this gene is rare in the precursor lesions of cSCC and in primary cSCC [19].

Increased cyclin D1 gene expression was observed in the precursor lesions of cSCC—AK and BD—and in primary cSCC. The cyclin D1 marker can be used as a prognostic marker in the diagnosis and treatment of cSCC [44,45,46,47,48].

Important roles in the differentiation of keratinocytes are played by the NOTCH1 gene and the NOTCH2 gene, which together make up the NOTCH (neurogenic locus notch homolog protein) family. These genes encode transmembrane receptors that are composed of an intracellular domain involved in transcription and an extracellular domain involved in binding to various ligands. The NOTCH1 gene has a direct involvement in the p53 pathway as it is involved in the cellular differentiation of keratinocytes. Given that NOTCH family gene defects are inactivation defects, they are thought to be tumor suppressor genes. The Wnt/β-catenin pathway is also affected by NOTCH1 gene mutation as this pathway is involved in oncogenesis [6,45,46,47]. The TP63 gene that encodes the p63 protein is also part of the category of p53 transcription factors; it is involved in the pathogenesis of cSCC and interferes with the processes of cell proliferation, differentiation, and senescence. The p63 protein is expressed by 70–100% of cSCC cases, including precursor lesions. [17]

**Figure 5:**
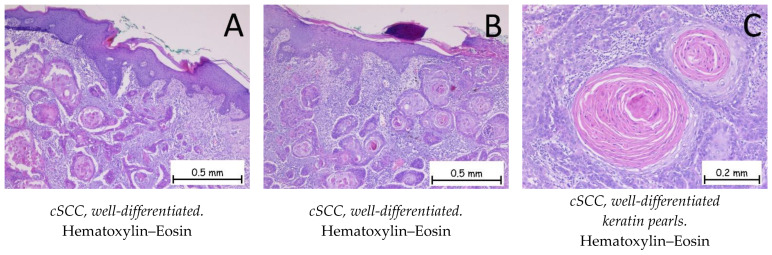
Cutaneous squamous cell carcinoma—well-differentiated. (**A**,**B**). Skin fragment covered by keratinized, stratified, squamous epithelium, presenting a tumoral proliferation in the underlying dermis composed of plaques of tumor cells with a squamous appearance. The plaques have a solid, compact appearance, with varied shapes and sizes, exhibiting marked keratinization phenomena. Adjacent to the tumoral plaques, there is an abundant polymorphic inflammatory infiltrate. At a higher magnification, the tumor cells have abundant, eosinophilic cytoplasm; enlarged, pleomorphic nuclei; mitotic figures, along with keratin pearl (**C**) formations.

**Figure 6:**
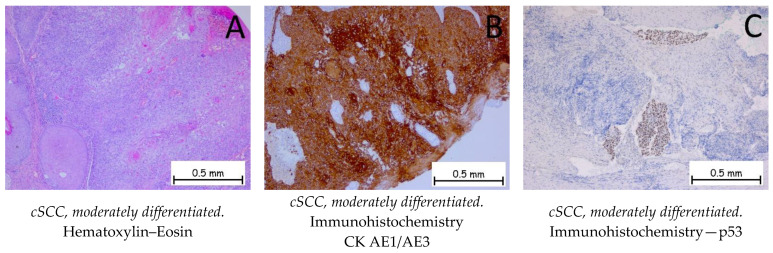
Cutaneous squamous cell carcinoma—moderately differentiated. (**A**). Proliferation of irregular patches and plaques with polygonal squamous cells, exhibiting marked cyto-nuclear atypia with rare occurrences of keratinization. Around the tumor, an abundant, mixed, inflammatory infiltrate is observed. The tumor cells are positive for anti-CK AE1/AE3 antibodies (**B**) and for anti-p53 antibodies (**C**).

**Figure 7:**
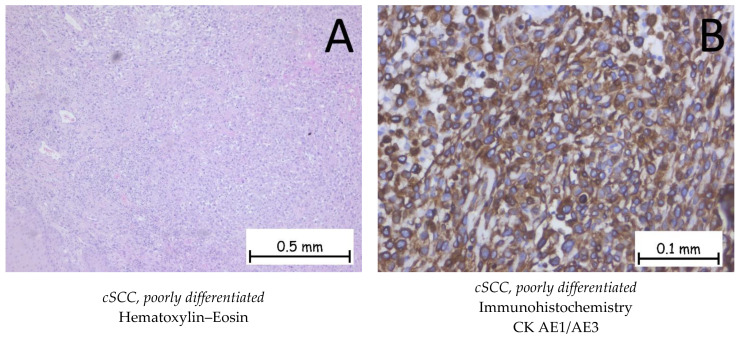
Cutaneous squamous cell carcinoma—poorly differentiated. (**A**). Solid proliferation of polygonal squamous cells with marked cyto-nuclear atypia. The cells have an abundant cytoplasm, which is either eosinophilic or pale, large pleomorphic nuclei with eosinophilic nucleoli, and numerous atypical mitoses. No keratinization phenomena are observed. (**B**). The tumor cells are positive for anti-CK AE1/AE3 antibodies.

*Squamous cell carcinoma* is represented by the proliferation of keratinocytes from the spinous (squamous) layer; it has different degrees of differentiation and goes beyond the basal membrane of the epidermis. Squamous cell carcinoma has an invasive character and is prone to metastasis and recurrence. It is classified into three grades, depending on the degree of squamous cell differentiation and the presence of keratinization: well-differentiated, moderately differentiated, and poorly differentiated (Figure 5, Figure 6 and Figure 7). The variants of squamous carcinoma are the acantholytic type—an invasive variant which forms pseudoglandular spaces and the phenomena of acantholysis; the spindle cell type—an invasive variant where the squamous cells lose their differentiation and the cells are spindle-shaped; the verrucous type—a well-differentiated variant of squamous carcinoma, which forms plaques of tumor cells, among which a chronic inflammatory infiltrate can be observed, with low metastatic potential; the adenosquamous type—a variant in which both glandular and squamous differentiations are present; the clear cell variant—a rare variant in which squamous cells show cytoplasmic vacuolations and areas of keratinization; the variant with sarcomatoid differentiation; the lymphoepithelioma-like variant; the pseudovascular variant; and the variant with osteoclast-like giant cells. Immunohistochemically, tumor cells are positive for anti-CK HMW, anti-CK AE1/AE3, anti-p-40, anti-p53, anti-CK5/6, and anti-MNF116 antibodies and negative for BerEP4. The differential diagnosis is made based on the presence of benign conditions, such as pseudoepitheliomatous hyperplasia, endophytic keratosis, scars, or adnexal tumors, for well-differentiated variants, or on the presence of melanoma or lymphoma for poorly differentiated variants. Pleomorphic dermal sarcoma should also be considered for differential diagnosis. In the case of cutaneous squamous cell carcinoma, it is necessary to carry out the differential diagnosis with a possible cutaneous metastasis of squamous cell carcinoma, such as in cases of squamous cell carcinoma of a pulmonary, head, or neck origin or a squamous cell carcinoma of the genital sphere. The genetic profile expresses mutations in the CXCL8, MMP1, HIF1A, ITGA6, and ITGA2 genes [1,48,49,50,51]. 

### 2.3. Differential Diagnosis of Cutaneous Squamous Cell Carcinoma

**Figure 8:**
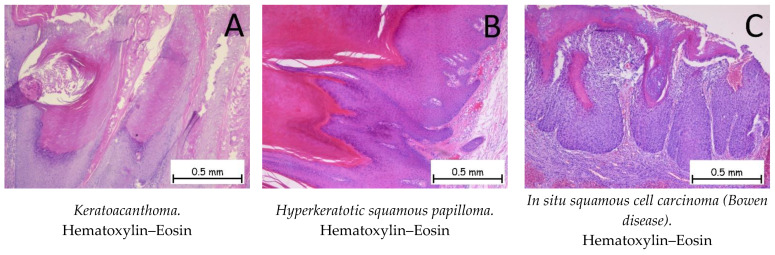
Keratoacanthoma, hyperkeratotic squamous papilloma, and in situ squamous cell carcinoma (Bowen disease). (**A**). Keratoacanthoma. Tegument covered by a keratinized stratified squamous epithelium, composed of squamous cells with minimal atypia, exhibiting pronounced orthokeratotic hyperkeratosis phenomena. (**B**). Hyperkeratotic squamous papilloma. Keratinized stratified squamous epithelium, without atypia, presenting a papillomatous formation on the surface covered by a keratinized stratified squamous epithelium with marked orthokeratotic hyperkeratosis, along with an acanthotic spinous layer. (**C**). In situ squamous cell carcinoma (Bowen disease). Skin fragment covered by intact keratinized stratified squamous epithelium, exhibiting marked dysplasia throughout its thickness. Tumor cells display pleomorphism, enlarged nuclei, and irregular nuclear membranes, with numerous mitoses. The tumor cells do not infiltrate the underlying dermis.

*Keratoacanthoma* represents a tumoral proliferation of squamous cells and is similar to the well-differentiated variant of cSCC. Keratoacanthoma has the ability to regress spontaneously. Depending on the evolutionary stage, three phases are distinguished: early lesions, characterized by a proliferation of squamous tumor cells arranged in the form of lobules with a pale cytoplasm; well-developed lesions, characterized by islands of tumor cells composed of squamous cells with a glassy cytoplasm, an altered nucleus–cytoplasm ratio in favor of the nucleus, and an inflammatory infiltrate between the tumor cells; and regressing lesions, characterized by the presence of keratin and the appearance of fibrosis in the dermis. The most recent AJCC manual and the WHO/WHO 2018 manual for the classification of skin tumors [1] consider keratoacanthoma to be a well-differentiated cSCC and assign it the code 8071/3. A well-differentiated or moderately differentiated microinvasive or invasive cSCC can often develop against a background of keratoacanthoma [1,52]. Figure 8A shows the histopathological appearance of keratoacanthoma.

Hyperkeratotic squamous papilloma is a benign, papillomatous tumor proliferation consisting of the epidermis showing acanthosis and the hyperkeratosis phenomena, which are generally associated with HPV infection [1,53]. Figure 8B shows the histological appearance of a hyperkeratotic squamous papilloma.

Squamous cell carcinoma in situ is also called Bowen disease (BD) and is represented by the tumor proliferation of dysplastic squamous cells throughout the thickness of the epidermis that do not go beyond its basement membrane (Figure 8C). Immunohistochemically, the cells are positive for anti-CK AE1/AE3, anti-p40, and anti-EMA antibodies, and negative for anti-Bcl-2 or anti-BerEP4 [1,48,53].

### 2.4. Differential Diagnosis with Other Non-Melanocytic Skin Conditions

**Figure 9:**
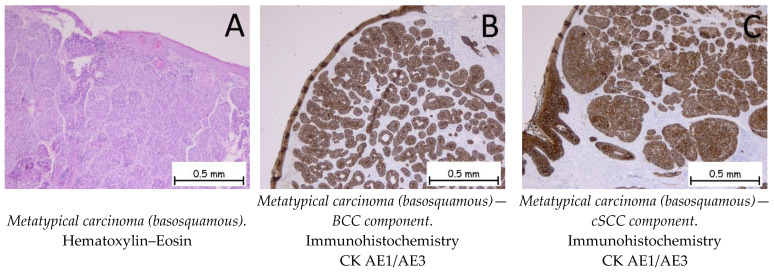
Metatypical carcinoma (basosquamous). (**A**). Tegument lined by a keratinized stratified squamous epithelium, exhibiting a tumoral proliferation consisting of two components: a predominantly squamous cell carcinoma component and a basal cell carcinoma component. (**B**). The tumor cells are positive for anti-CK AE1/AE3 antibodies in the BCC component. (**C**). The tumor cells are positive for anti-CK AE1/AE3 antibodies in the cSCC component.

*Metatypical (basosquamous) carcinoma* shows a proliferation of tumor cells that have microscopic features of both basaloid and squamous cells (Figure 9). Immunohistochemically, both cell populations are positive for anti-CK AE1/AE3 antibodies. The squamous cell carcinoma component is positive for anti-EMA and anti-p40 antibodies, and the basal cell carcinoma component is positive for anti-Bcl-2 and anti-BerEP4 antibodies. The differential diagnosis is made with squamous carcinomas with basaloid differentiations, as well as with the independent components that make up the metatypical carcinoma by means of immunohistochemistry [1,54].

**Figure 10:**
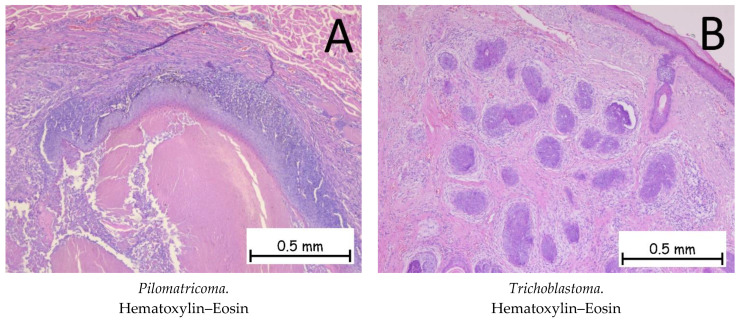
Pilomatricoma and trichoblastoma. (**A**). Pilomatricoma—tumoral proliferation consisting of islands of basaloid cells without atypia, occasionally flattened, and focal areas completely devoid of cells, replaced by cells with indistinct contours, intensely eosinophilic cytoplasm, and inconspicuous nuclei (phantom cells) extending towards the center of the lesion. (**B**). Trichoblastoma. Tumoral proliferation composed of nodules of varying sizes with cells that have basophilic cytoplasm and are monomorphic and without cyto-nuclear atypia. The surrounding stroma has a fibromyxoid appearance.

*The pilomatricoma* (Figure 10A) is composed of basaloid cells with the presence of mitoses and phantom cells (polygonal-shaped cells represented by cell reminiscences with a centrally located, optically clear zone). Immunohistochemically, tumor cells are positive for anti-β-catenin antibodies. The differential diagnosis is made with basal cell carcinoma, where the presence of phantom cells directs the diagnosis towards pilomatrixoma [1,55].

*Trichoblastoma* (Figure 10B) represents a benign proliferation of tumor cells at the level of the hair follicles. The histopathological appearance is characterized by the presence of basaloid cells surrounded by a fibrous stroma. In the case of trichoblastoma, the presence of papillary bodies, formed by basaloid cells and fibroblasts, is characteristic. Immunohistochemically, the cells are positive for anti-Bcl-2 antibodies. The differential diagnosis is made with basal cell carcinomas, where the presence of papillary bodies is absent and the stroma around the tumor cells differs. The presence of mitoses is much more expressed in BCCs [1,56].

### 2.5. Differential Diagnosis with Other Skin Malignancies

**Figure 11:**
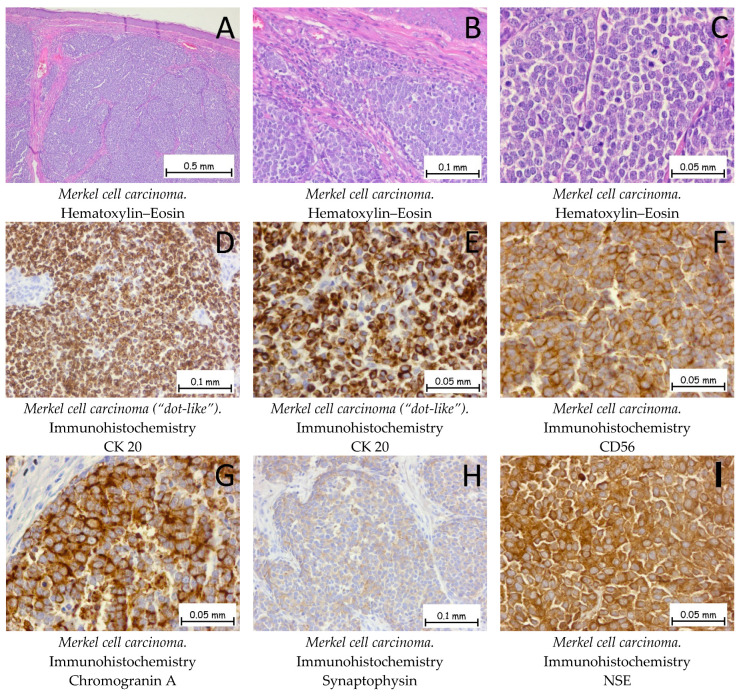
Merkel cell carcinoma. (**A**,**B**). The tumor formation exhibits a solid architecture characterized by plaques and trabeculae of monomorphic cells, small to medium in size. The tumor cells do not show epidermotropism, without an ulceration of the epidermis. Between the tumoral plaques, there is a moderate chronic inflammatory infiltrate. (**C**). At a higher magnification, the tumoral cells have a hyperchromatic, centrally located nucleus with granular and dispersed chromatin, along with an elevated mitotic index. Tumor cells are positive for anti-CK-20 antibodies (**D**,**E**), anti-CD56 antibodies (**F**), anti-Chromogranin A antibodies (**G**), anti-Synaptophysin antibodies (**H**), and anti-NSE antibodies (**I**).

*Merkel cell carcinoma (MCC)* is a very rare form of cutaneous neoplasia with a neuroendocrine origin, which has been described as one of the most aggressive cutaneous malignancies. Histopathologically, the Merkel cell carcinoma is a tumor proliferation consisting of small, monomorphic, basophilic tumor cells with scanty, pale cytoplasm; round nuclei with granular, dispersed chromatin; few or absent nucleoli; and numerous mitotic figures. Tumor proliferation may occupy the entire dermis with an extension into the hypodermis, and the epidermis is generally intact, with 30% of the tumors showing epidermotropism and pagetoid migration (Figure 11). The origin of MCCs is still disputed; currently under discussion is whether the origin of the tumoral cells is epithelial or non-epithelial. The main oncogenic factor discussed with regard to MCCs is the Merkel cell polyomavirus, which is found in around 80% of cases [57]. In his article, Kervarrec T. emphasized that, for infections, the virus enters the cell cycle during the mitotic phase and that Merkel cells are post-mitotic [58]. Another hypothesis that may lead to a non-epithelial origin of the cell considers the fact that tumor cells often express B-cell markers. Regardless of the origin of the cell, MCC remains a high-risk skin malignancy with a bad prognosis [57,58,59]. Squamous cell carcinoma in situ (Bowen disease) can be associated with an underlying MCC. Immunohistochemically, the tumor cells are positive for anti-CK20 and anti-CK AE1/AE3 antibodies with a characteristic “dot-like” pattern. Neuroendocrine markers, such as Chromogranin A, Synaptophysin, CD56, and NSE (neuron-specific enolase), are also positive. The differential diagnoses are melanoma, cutaneous lymphoma, and extramammary Paget’s disease (if the epidermis is affected by pagetoid migration). Tumor cells are negative for immunolabeling with anti-S100 and anti-LCA antibodies. Genetic profiling for Merkel cell carcinomas shows mutations in the TP53 and RB1 genes [1,60,61].

**Figure 12:**
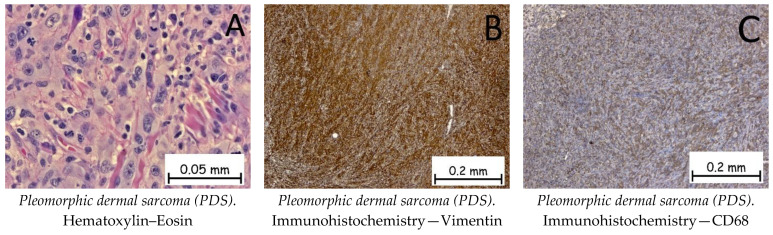
Pleomorphic dermal sarcoma (PDS). (**A**). Tumoral proliferation with solid architecture composed of pleomorphic tumoral cells with epithelioid or fusiform appearances, vesicular nuclei, and prominent nucleoli admixed with multinucleated giant cells, along with numerous mitotic figures. Tumor cells are positive for anti-Vimentin antibodies (**B**) and anti-CD68 antibodies (**C**).

*Pleomorphic dermal sarcoma* is a malignant mesenchymal tumor of primary origin at the level of the dermis. The tumor cells are epithelioid or fusiform in appearance and arranged in the form of beaches, with multiple nuclei, hyperchromasia, and a high number of mitoses (Figure 12). Immunohistochemically, the tumor cells are positive for anti-vimentin antibodies, anti-CD10 antibodies, and anti-CD68 antibodies, and negative for anti-SOX-10 and CK AE1/AE3 antibodies (Figure 12). The differential diagnosis of pleomorphic dermal sarcoma is made with epithelioid melanoma and poorly differentiated cutaneous squamous carcinoma, where immunohistochemistry can establish the diagnosis of certainty by means of S100 or EMA markers [1,62].

**Figure 13:**
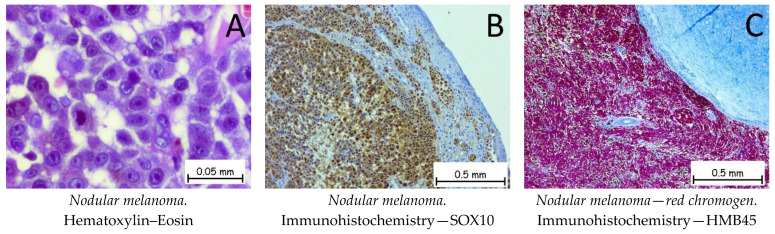
Nodular melanoma. (**A**). Tumoral cells with epithelioid appearances with abundant eosinophilic cytoplasm and vesicular nuclei and prominent nucleoli. Tumor cells are positive for anti-SOX10 antibodies (**B**) and anti-HMB45 (red chromogen) antibodies (**C**).

*Nodular melanoma* (Figure 13) is the most common malignant, melanocytic skin tumor. The tumor cells can be epithelioid, fusiform, or mixed in appearance. The epithelioid cells have abundant eosinophilic cytoplasm with enlarged, round–oval nuclei. Immunohistochemically, the tumor cells are positive for anti-melan-A, anti-HMB45, anti-S100, PRAME, and anti-SOX10 antibodies. The differential diagnosis should be made with cSCC if the tumor cells do not show melanic pigments. Immunohistochemically, the tumor cells are positive for melanocyte markers and negative for CK AE1/AE3 and for CD3 or LCA. The genetic profile for a nodular melanoma shows mutations in the BRAF, CDKN2A, TP53, or TERT genes [1,63,64,65].

Even though the treatment of NMSCs is currently based on surgical and oncological treatments and seems easily accessible by many patients, the COVID-19 pandemic had a very important role in the dermatological diagnostics, histopathological diagnostics, and treatment for these patients. This has resulted in more advanced stages, with less prevention, and a decrease in the quality of life of these patients, and is directly related to their satisfaction and outcome [66,67].

## 3. Materials and Methods

We performed a quantitative and descriptive study, with reference to the recent literature, to provide a concise view of the pathophysiology, histopathological diagnostics, and differential diagnostics of BCC and cSCC. The study was based on an update of the newest literature findings, in combination with the presentation of real-life diagnostics of BCC and cSCC, emphasizing the morphological characteristics and the immunohistochemical profiles of NMSCs on real-life histopathological slides. 

The quantitative study was based on the scanning of the histological slides of patients who had undergone a surgical excision of a cutaneous tumoral process in the General Surgery Department of the Mures Clinical County Hospital. The histopathological diagnostics were established in the Clinical Pathology Department of our hospital, and the cases were inspected by a pathology consultant and three young pathologists. The period in which the cases were diagnosed was 2020–2022. The inclusion criteria for the cases were based on the histopathological diagnoses of basal cell carcinoma, cutaneous squamous cell carcinoma, and the differential diagnoses of skin pathologies, including different subtypes of BCC, metatypical carcinoma, keratoacanthoma, hyperkeratotic squamous papilloma, Bowen disease, trichoblastoma, pilomatricoma, Merkel cell carcinoma, pleomorphic dermal sarcoma, and melanoma.

The excised tissue specimens underwent preservation in a 4% formaldehyde solution derived from a 10% neutral buffered formalin. After grossing, the included tissue specimens experienced dehydration through immersion in ethyl alcohol with progressive ascending concentrations of 70° and 96° and in absolute ethyl alcohol. Following dehydration, the tissue specimens were embedded in paraffin to form the paraffin blocks. Sectioning through the paraffin blocks was achieved using a semi-automated microtome and microtome blades, and the resulting sections were mounted on histological glass slides. Deparaffination was executed using xylene, and the subsequent tissue hydration involved a series of decreasing alcohol concentrations. An automated stainer was utilized for Hematoxylin–Eosin staining. For immunohistochemistry, after sectioning the paraffin blocks, the sections were mounted on special immunohistochemistry frosted slides. For the immunohistochemistry reactions, we used the manual and automated methods, using the protocols provided by each manufacture of the antibodies. The evaluation of the slides transpired under a light microscope, facilitated by a Zeiss Axio Lab A1 Microscope and Zeiss ZenPro 3.2 Software for photo capturing, editing, and adding the scale on each picture; the process was conducted by four pathologists. Informed consent was obtained from all the subjects involved in the study.

## 4. Conclusions

Our study showed the importance of a correct histopathological diagnosis, which had a direct impact on the appropriate treatment for each patient. The study highlighted the histopathological and morphological characteristics of NMSCs and the precursor lesions in HE and the immunohistochemical profiles for the lesions that could make diagnoses difficult. The differential diagnoses of BCC and cSCC are necessary and should be based on the morphologic features and immunohistochemical profiles of each individual lesion. The originality lies in the iconographic presentation of the benign and malignant lesions with which the differential diagnoses of BCC and cSCC must be made.

To establish a histopathological diagnosis, it is necessary for all the histopathological parameters of the tumor formations to be analyzed, both by Hematoxylin–Eosin staining and by immunohistochemical reactions. Thus, a definitive histopathological diagnosis can be formulated, with a direct impact on the appropriate treatment for each patient. The differential diagnoses of BCC and cSCC must be made correctly, based on the morphological characteristics and immunohistochemical profiles of each individual lesion.

Even though digital pathology is advancing quickly, a histopathological diagnosis based on the morphological characteristics and immunohistochemical reactions remains the most important tool to establish the correct treatment and management of a case by the multidisciplinary team in order to achieve the best outcomes for the patients and thereby to increase their quality of life.

## Figures and Tables

**Table 1:** Main characteristics and classifications of BCC and cSCC [1,2,14,30,31,33,34,35].

	Basal Cell Carcinoma (BCC)	Cutaneous Squamous Cell Carcinoma (cSCC)
**Origin**	Develops from the cells of the basal layer of the epidermis but can also derive from the level of the hair follicle BCC consists of a tumoral proliferation of basaloid cells with reduced cytoplasm and hyperchromic nuclei, surrounded by a fibromyxoid stroma	Develops from cells in the spinous layer of the epidermis
**Precursor lesions**	No precursor lesions	Bowen disease (squamous cell carcinoma in situ—BD) Actinic keratosis (AK) Keratoacanthoma (KA)
**Histopathological classification**	Classification of subtypes by recurrence risk: Low risk: Nodular BCC Superficial multicentric BCC Pigmentated BCC Cystic BCC Fibroepithelial BCC (Pinkus) High risk: Metatypical BCC Morpheipform BCC Infiltrative BCC BCC with sarcomatoid differentiation Micronodular BCC	Classification by recurrence or metastasizing risk: Low risk: Tumor size below 20 mm Tumor thickness below 2 mm Clark level less than or equal to IV No or a low degree of tumor budding Without perineural or lympho-vascular invasions Types associated with a low risk: classical type, keratoacanthoma, verrucous type, acantholytic type, or clear cell type High risk: Tumor size greater than or equal to 20 mm Tumor thickness greater than or equal to 2 mm Clark level V A high degree of tumor budding; perineural or lympho-vascular invasions Types associated with a high risk: spindle cell type, adenosquamous type, or pseudovascular type The classical, infiltrative type can also be classified into high, moderate, or low cSCCs, depending on the degree of differentiation of the tumoral cells

## Data Availability

The data presented in this study are available on request from the corresponding author.
